# Supplementary material for: Pyruvate Dehydrogenase Kinase 1 inhibition mediated oxidative phosphorylation enhancement in cartilage promotes osteoarthritis progression
Source: BMC Musculoskelet Disord. 2023 Jul 20;24:597. doi: 10.1186/s12891-023-06585-6 (PMC10357736; doi:10.1186/s12891-023-06585-6)
Supplement: Supplementary file 1 — Additional file 1. [file 12891_2023_6585_MOESM1_ESM.zip › KEGG.Gsea.1653622432464/KEGG_BUTANOATE_METABOLISM.html]

Details for gene set KEGG\_BUTANOATE\_METABOLISM[GSEA]

|  || Dataset | 55457\_collapsed\_to\_symbols.cls.cls #OA\_versus\_normal.cls.cls #OA\_versus\_normal\_repos |
| Phenotype | cls.cls#OA\_versus\_normal\_repos |
| Upregulated in class | OA |
| GeneSet | KEGG\_BUTANOATE\_METABOLISM |
| Enrichment Score (ES) | 0.3774251 |
| Normalized Enrichment Score (NES) | 1.3790748 |
| Nominal p-value | 0.07621247 |
| FDR q-value | 0.12613764 |
| FWER p-Value | 0.971 |
Table: GSEA Results Summary

  

Fig 1: Enrichment plot: KEGG\_BUTANOATE\_METABOLISM      
 Profile of the Running ES Score & Positions of GeneSet Members on the Rank Ordered List

  

| SYMBOL | TITLE | RANK IN GENE LIST | RANK METRIC SCORE | RUNNING ES | CORE ENRICHMENT || 1 | PDHB | pyruvate dehydrogenase E1 subunit beta [Source:HGNC Symbol;Acc:HGNC:8808] | 149 | 0.862 | 0.0783 | Yes |
| 2 | ALDH2 | aldehyde dehydrogenase 2 family member [Source:HGNC Symbol;Acc:HGNC:404] | 1003 | 0.517 | 0.0659 | Yes |
| 3 | ACAT1 | acetyl-CoA acetyltransferase 1 [Source:HGNC Symbol;Acc:HGNC:93] | 1017 | 0.513 | 0.1185 | Yes |
| 4 | ALDH3A2 | aldehyde dehydrogenase 3 family member A2 [Source:HGNC Symbol;Acc:HGNC:403] | 1263 | 0.465 | 0.1479 | Yes |
| 5 | ALDH5A1 | aldehyde dehydrogenase 5 family member A1 [Source:HGNC Symbol;Acc:HGNC:408] | 1661 | 0.405 | 0.1593 | Yes |
| 6 | HMGCL | 3-hydroxy-3-methylglutaryl-CoA lyase [Source:HGNC Symbol;Acc:HGNC:5005] | 1703 | 0.398 | 0.1977 | Yes |
| 7 | ACSM3 | acyl-CoA synthetase medium chain family member 3 [Source:HGNC Symbol;Acc:HGNC:10522] | 1835 | 0.380 | 0.2271 | Yes |
| 8 | HMGCS2 | 3-hydroxy-3-methylglutaryl-CoA synthase 2 [Source:HGNC Symbol;Acc:HGNC:5008] | 1888 | 0.374 | 0.2621 | Yes |
| 9 | ECHS1 | "enoyl-CoA hydratase, short chain 1 [Source:HGNC Symbol;Acc:HGNC:3151]" | 1925 | 0.369 | 0.2978 | Yes |
| 10 | ALDH7A1 | aldehyde dehydrogenase 7 family member A1 [Source:HGNC Symbol;Acc:HGNC:877] | 2158 | 0.343 | 0.3156 | Yes |
| 11 | PDHA1 | pyruvate dehydrogenase E1 subunit alpha 1 [Source:HGNC Symbol;Acc:HGNC:8806] | 2433 | 0.310 | 0.3266 | Yes |
| 12 | GAD1 | glutamate decarboxylase 1 [Source:HGNC Symbol;Acc:HGNC:4092] | 2534 | 0.299 | 0.3501 | Yes |
| 13 | ALDH9A1 | aldehyde dehydrogenase 9 family member A1 [Source:HGNC Symbol;Acc:HGNC:412] | 2795 | 0.270 | 0.3580 | Yes |
| 14 | OXCT1 | 3-oxoacid CoA-transferase 1 [Source:HGNC Symbol;Acc:HGNC:8527] | 2897 | 0.261 | 0.3774 | Yes |
| 15 | OXCT2 | 3-oxoacid CoA-transferase 2 [Source:HGNC Symbol;Acc:HGNC:18606] | 4035 | 0.152 | 0.3050 | No |
| 16 | ACSM2A | acyl-CoA synthetase medium chain family member 2A [Source:HGNC Symbol;Acc:HGNC:32017] | 4080 | 0.148 | 0.3170 | No |
| 17 | HADHA | hydroxyacyl-CoA dehydrogenase trifunctional multienzyme complex subunit alpha [Source:HGNC Symbol;Acc:HGNC:4801] | 4163 | 0.141 | 0.3253 | No |
| 18 | BDH2 | 3-hydroxybutyrate dehydrogenase 2 [Source:HGNC Symbol;Acc:HGNC:32389] | 4247 | 0.133 | 0.3328 | No |
| 19 | ACSM5 | acyl-CoA synthetase medium chain family member 5 [Source:HGNC Symbol;Acc:HGNC:26060] | 4976 | 0.074 | 0.2840 | No |
| 20 | HADH | hydroxyacyl-CoA dehydrogenase [Source:HGNC Symbol;Acc:HGNC:4799] | 5088 | 0.065 | 0.2822 | No |
| 21 | ACAT2 | acetyl-CoA acetyltransferase 2 [Source:HGNC Symbol;Acc:HGNC:94] | 6291 | -0.027 | 0.1915 | No |
| 22 | ALDH1B1 | aldehyde dehydrogenase 1 family member B1 [Source:HGNC Symbol;Acc:HGNC:407] | 6551 | -0.046 | 0.1761 | No |
| 23 | ABAT | 4-aminobutyrate aminotransferase [Source:HGNC Symbol;Acc:HGNC:23] | 6804 | -0.063 | 0.1632 | No |
| 24 | EHHADH | enoyl-CoA hydratase and 3-hydroxyacyl CoA dehydrogenase [Source:HGNC Symbol;Acc:HGNC:3247] | 7968 | -0.153 | 0.0887 | No |
| 25 | ACSM1 | acyl-CoA synthetase medium chain family member 1 [Source:HGNC Symbol;Acc:HGNC:18049] | 8210 | -0.172 | 0.0879 | No |
| 26 | AKR1B10 | aldo-keto reductase family 1 member B10 [Source:HGNC Symbol;Acc:HGNC:382] | 8420 | -0.187 | 0.0912 | No |
| 27 | ACADS | acyl-CoA dehydrogenase short chain [Source:HGNC Symbol;Acc:HGNC:90] | 9105 | -0.240 | 0.0630 | No |
| 28 | PDHA2 | pyruvate dehydrogenase E1 subunit alpha 2 [Source:HGNC Symbol;Acc:HGNC:8807] | 9124 | -0.241 | 0.0868 | No |
| 29 | GAD2 | glutamate decarboxylase 2 [Source:HGNC Symbol;Acc:HGNC:4093] | 9249 | -0.252 | 0.1034 | No |
| 30 | BDH1 | 3-hydroxybutyrate dehydrogenase 1 [Source:HGNC Symbol;Acc:HGNC:1027] | 10049 | -0.326 | 0.0754 | No |
| 31 | AACS | acetoacetyl-CoA synthetase [Source:HGNC Symbol;Acc:HGNC:21298] | 10226 | -0.344 | 0.0975 | No |
| 32 | HMGCS1 | 3-hydroxy-3-methylglutaryl-CoA synthase 1 [Source:HGNC Symbol;Acc:HGNC:5007] | 10829 | -0.408 | 0.0933 | No |
| 33 | L2HGDH | L-2-hydroxyglutarate dehydrogenase [Source:HGNC Symbol;Acc:HGNC:20499] | 12208 | -0.648 | 0.0538 | No |
Table: GSEA details [plain text format]

  

Fig 2: KEGG\_BUTANOATE\_METABOLISM      
 Blue-Pink O' Gram in the Space of the Analyzed GeneSet

  

Fig 3: KEGG\_BUTANOATE\_METABOLISM: Random ES distribution      
 Gene set null distribution of ES for **KEGG\_BUTANOATE\_METABOLISM**
